# Implant of ATOMS^®^ system for the treatment of postoperative male stress urinary incontinence: results of a single centre

**DOI:** 10.1590/S1677-5538.IBJU.2018.0171

**Published:** 2019

**Authors:** Alessandro Giammò, Enrico Ammirati, Annarita Tullio, Gianni Bodo, Alberto Manassero, Paolo Gontero, Roberto Carone

**Affiliations:** 1 Department of Neuro-Urology, CTO - Spinal Unit, Città della Salute e della Scienza di Torino, Turin, Italy;; 2 Department of Urology, Molinette Hospital, Città della Salute e della Scienza di Torino, Turin, Italy;; 3 Hygiene and Clinical Epidemiology Unit, S. Maria della Misericordia University Hospital of Udine, Udine, Italy

**Keywords:** Suburethral Slings, Urinary Incontinence, Surgical Procedures, Operative

## Abstract

**Purpose:**

The aim of our study is to evaluate the efficacy and safety of ATOMS^®^ system for the treatment of postoperative male stress urinary incontinence (SUI).

**Materials and methods:**

We retrospectively evaluated all patients treated at our institution for postoperative male SUI with ATOMS^®^ implant. We excluded patients with low bladder compliance (< 20 mL / cmH_2_O), uncontrolled detrusor overactivity, detrusor underactivity (BCI < 100), urethral or bladder neck stricture and low cystometric capacity (< 200 mL).

**Results:**

From October 2014 to July 2017 we treated 52 patients, mean age 73.6 years. Most of them (92.3%) had undergone radical prostatectomy, 3.85% simple open prostatectomy, 3.85% TURP; 28.8% of patients had undergone urethral surgery, 11.5% adjuvant radiotherapy; 57.7% had already undergone surgical treatment for urinary incontinence. The average24 hours pad test was 411.6 g (180 – 1100). The mean follow-up was 20.1 months (8.1 – 41.5) 30.8% of patients were dry, 59.6% improved ≥ 50%, 7.7% improved < 50% and 1.9% unchanged. In total 73.1% reached social continence. There was a significant reduction of the 24 hours pad test and ICIQ - UI SF scores (p < 0.01).

In the postoperative follow-up we detected complications in 8 patients (19%): 5 cases of displacement of the scrotal port, in 2 cases catheterization difficulties, one case of epididimitis and concomitant superficial wound infection; no prosthesis infection, nor explants.

Radiotherapy, previous urethral surgery,previous incontinence surgery were not statistically related to social continence rates (p 0.65;p 0.11;p 0.11).

**Conclusions:**

The ATOMS^®^ system is an effective and safe surgical treatment of mild and moderate male postoperative SUI with durable results in the short term.

## INTRODUCTION

Radical prostatectomy (RP) is considered the main cause of male stress urinary incontinence (SUI) with variable incidence between 4% and 40% (1, 2). The main predictive factors for postoperative stress incontinence are age, body mass index, comorbidity index, presence of low urinary tract symptoms (LUTS), prostate volume and surgeon expertise (2, 3). Surgical treatment of benign prostatic hyperplasia (BPH) has a lower incidence of postoperative SUI, ranging between 1.9 – 2.2% (4). Most patients are offered pelvic floor muscle exercise, that improves early continence rates but not long term continence rates (5). When conservative treatments have failed, patients are usually offered surgical treatments after stabilization at 12 – 18 months. The artificial urinary sphincter (AUS) is still considered the gold standard for the treatment of male SUI with dry rates up to 86% (6), but high costs, postoperative complication and re - operation rates of the artificial urinary sphincter (AUS) led to the development of alternative systems. The continence results of sling systems vary around 50%, depending on the definition for therapeutic success (7). Most slings are not adjustable and some can be adjusted after implantation only with a small surgical procedure. The ATOMS^®^ system was created to overcome this issues and was introduced in Europe in 2008. It is an extra urethral bulking system in form of a hydraulic sphincter cushion support made of three parts: two polypropylene mesh arms, a cushion and a titanium port. Compression can be modulated with office - based filling procedures. Unlike the artificial sphincter, which encircles the urethra circumferentially by interfering with venous blood flow and predisposing the urethra to atrophy and erosion, the ATOMS^®^ system only compresses the ventral portion of the urethra, leaving the dorsal and lateral blood flow intact (7, 8).

The aim of this work is to evaluate mid - term efficacy and safety of ATOMS^®^ system for the treatment of postoperative male SUI.

## MATERIALS AND METHODS

This retrospective study was conducted between October 2014 and July 2017. We enrolled all consecutive ATOMS^®^ implants at our center. All patients were clinically diagnosed with non - neurogenic postoperative stress urinary incontinence. Before surgical treatment each patient underwent anamnestic data collection, 24 hours pad test and pad count, physical examination, urodynamic evaluation, ICIQ - UI SF questionnaire. We evaluated the severity of urinary incontinence referring to the 24 hours pad test: no incontinence (0 pads / day), mild incontinence (< 200 g / day), moderate incontinence (200 – 400 g / day), severe incontinence (> 400 g / day). We excluded and did not implant patients with low bladder compliance (< 20 mL / cmH_2_O), uncontrolled detrusor overactivity (DO), detrusor underactivity (BCI < 100), urethral or bladder neck stricture and low cystometric capacity (< 200 mL). All patients were implanted with ATOMS^®^ system by a single surgeon. Patients were followed up with a first clinical visit at 1 month after the intervention and the device was refilled in cases of persistent stress urinary incontinence. There was not a fixed schedule for subsequent follow-up visit. At the last follow-up, all patients underwent a follow-up physical examination, 24 hours pad test and pad count, ICIQ - UI SF and PGI - I questionnaire. The primary objective of this study was to evaluate the efficacy, safety and patient satisfaction of the ATOMS^®^ system from the last follow-up.

### Statstical analysis

The population has been described through descriptive analyses. For numerical variables, mean, median, minimum and maximum values, SD, IQR have been calculated. The normality of the distribution has been checked with the Kolmogorov - Smirnov test. As numerical variables were not normally distributed, we considered median ± intrerquartile range and we applied non parametric tests. For categorical variables have been calculated absolute and relative frequency distributions and double entry tables have been created. To evaluate the relationship between categorical variables, we applied Fisher exact test, being the size of some cells less than 5. To evaluate the relationship between outcome and predictors, we applied the calculation of 95% RR. The non parametric Wilcoxon Signed Ranks test for paired samples has been used to compare pre and post operative numerical non - normally distributed variables. All statistical analyses were performed using R 3.4.2 software. The significance level was set at 0.05.

## SURGICAL TECHNIQUE

**Figure 1:**
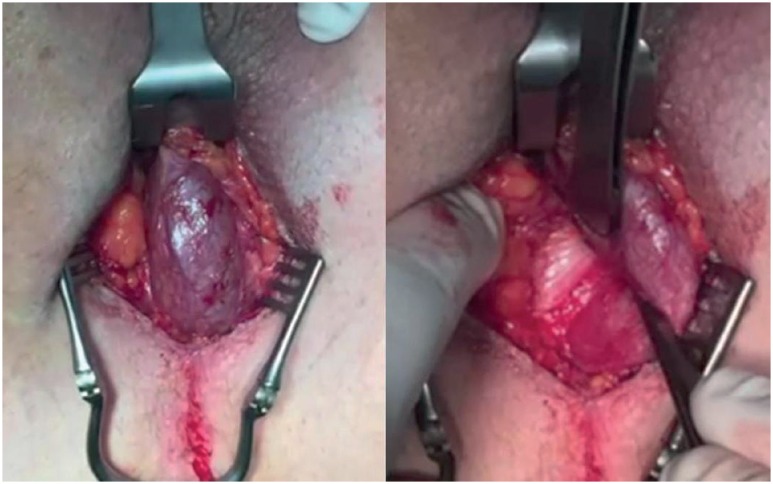
Dissection plan prepared until fibers of the bulbospongious muscle are encountered; lateral dissection plane evidencing the base of the crura.

**Figure 2:**
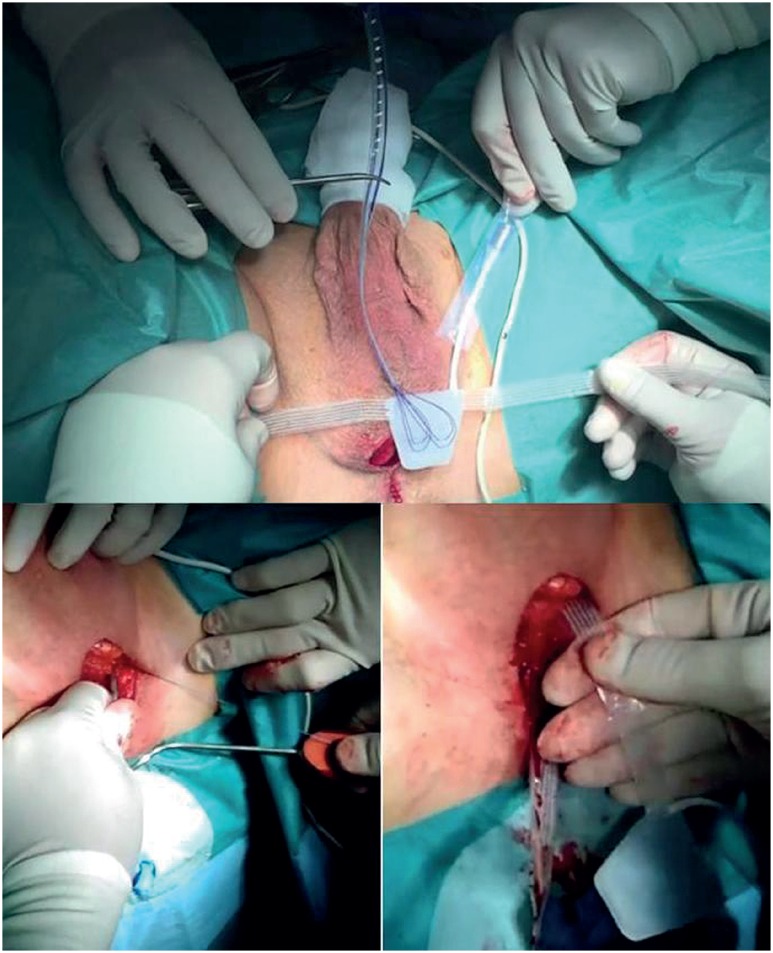
Outside-in transobturator passage of the arms with a specific tunneler.

**Figure 3:**
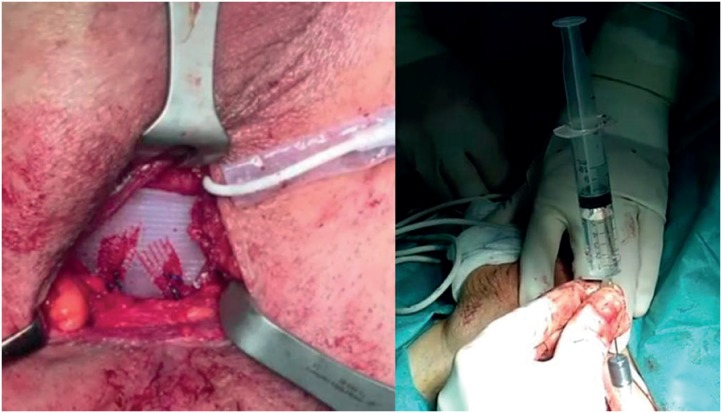
Position of the cushion after fixation of the arms; filling of the cushion with saline solution.

**Figure 4:**
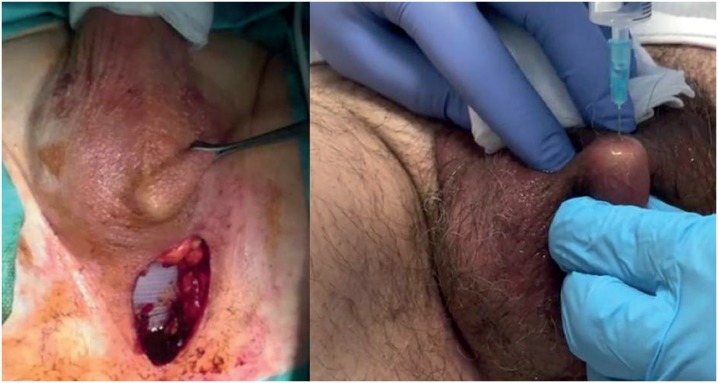
Dartos scrotal pouch that accommodates the port; refill at clinical visit.

The ATOMS^®^ system implant is fully standardized and facilitated by the single - piece configuration of the device. The patient is positioned in a lithotomy position with excellent perineum exposure and adequate flexion of the hips. A 14 French urethral catheter is positioned. After a vertical incision of 4 – 5 cm, the dissection plan is prepared until fibers of the bulbospongious muscle are encountered, maintaining them intact. Laterally the dissection plane follows the muscle fibers until the base of the crura. To reduce postoperative pain, it is important to avoid damaging the posterior scrotal nerves (Figure-1). A good preparation of the ischiorectal fossa is important for the placement of the implant fixation arms. With a specific curved tunneller, the arms of the sling are positioned bilaterally with an outside - in transobturator passage (Figure-2). Then the silicon cushion is fixed to the arms with two non - absorbable monofilament sutures. After removing the air present in the system, it is filled with saline solution until reaching ambient pressure (Figure-3). Depending on the degree of incontinence and detrusor contractility, the system can be filled further with one mL of saline solution. Finally, a dartos scrotal pouch is created to accommodate the port (Figure-4). A double - layer suturing closure guarantees a low risk of infection and extrusion of the device. There is no need, according to standardized technique, to place any drainage.

## RESULTS

We treated 52 male patients with ATOMS^®^ system. The average age was 73.6 years (median 73.7, range 58.9 – 84.3). In this population, 44 (84.6%) patients had undergone open radical open prostatectomy, 3 (5.8%) laparoscopic radical prostatectomy, 1 robot - assisted radical prostatectomy (1.9%), 2 (3.85%) patients transvesical simple open prostatectomy and 2 (3.85%) transurethral resection of the prostate (TURP). The average interval between the intervention and ATOMS^®^ implant was 6.84 years on average (range 1.41 – 16.77). Among the patients who underwent radical prostatectomy, 42 (80.8%) had a bilateral nerve - sparing (NS) procedure and 4 (7.7%) an unilateral NS procedure; 8 had undergone urethrotomy for an urethral stricture, 7 had undergone vesicoureteral anastomosis resection; at pathology report all patients were diagnosed with prostate adenocarcinoma, clinical and pathological stage are reported in Table-1. Six patients had undergone adjuvant radiotherapy, 5 were on hormone therapy with LHRH analogue / LHRH antagonist. All patients were in complete remission from cancer disease, in cases of radical prostatectomy with undetectable PSA. Preoperative urodynamic testing demonstrated in all patients a good bladder compliance (> 20 cmH_2_O / mL), absence of DO and the presence of urodynamic stress urinary incontinence with mean VLPP 64.79 cmH_2_O (median 62.5, range 32 – 105). The average 24 hours pad test was 411.6 g (median 370, range 180 – 1100 g). The average number of pads used was 4.23 (median 4, range 2 – 8) 3 patients (5.8%) were suffering from mild incontinence, 40 patients (76.9%) moderate incontinence, 9 patients (17.3%) severe incontinence. Thirty patients (57.7%) had already undergone surgical treatment for urinary incontinence: in 26 cases a ProACT device, in one case an artificial sphincter (FlowSecure), in two cases both a ProACT device then an artificial sphincter (AMS800), in one case both an intraurethral bulking therapy and subsequently a ProACT. Nineteen patients had undergone pelvic floor rehabilitation. Thirteen patients were treated with antimuscarinic oral therapy for OAB syndrome, without evidence of DO at preoperative urodynamic testing; 11 patients were treated with duloxetine without sustainable results.

**Table 1:** Clinical and pathological stage.

Clinical stage	Patients
cT1c N0/N× M0	38 (73.1%)
cT2a N0/N× M0	8 (15,4%)
cT2b N0/N× M0	3 (5.8%)
cT2c N0/N× M0	1 (1.9%)
cT3b N0/N× M0	2 (3.8%)
**Patological stage**	**Patients**
pT2a N0 M0	14 (26.9%)
pT2b N0 M0	20 (38.5%)
pT2c N0 M0	8 (15.4%)
pT3a N0 M0	5 (9.6%)
pT3b N0 M0	4 (7.7%)
pT3b N1 M0	1 (1.9%)
**Pathological Gleason score**	**Patients**
Gleson 6	22 (42.3%)
Gleason 7 (3+4)	18 (34.6%)
Gleason 7 (4+3)	10 (19.2%)
Gleason 8	2 (3.9%)

The average surgical time was 51.0 minutes (median 49, range 40 – 84). The mean follow-up was 22.1 months (median 20.7, range 8.1 – 41.5). One patient was lost at follow-up. The average number of fillings was 1.55 (median 1, range 0 – 8) with an average filling volume of 10.7 mL (median 9, range 0 – 28). In the immediate postoperative time, there were no severe complications (Clavien - Dindo ≥ 2), only 6 cases of scrotal pain and numbness persisting at 4 weeks after implant, solved with oral anti - inflammatory therapy, and 2 cases of acute postoperative retention, treated with temporary deflate of the cushion.

In the postoperative follow-up we detected complications in 8 patients (19%): 5 cases of displacement of the scrotal port (with surgical replacement in all cases), in 2 cases catheterization difficulties and difficulty to deflate the device, one case of epididimitis and concomitant superficial wound infection, treated with prolonged antibiotic therapy. There has been no prosthesis infection, nor explantations.

At the last follow-up available, 16 patients (30.8%) were completely dry, 31 (59.6%) improved ≥ 50%, 4 (7.7%) improved < 50% and 1 (1.9%) did not change. In total 38 patients (73.1%,) reached social continence (use of 0 or 1 security pad / day) (Table-2) (all p < 0.05, Fisher's exact test). We had a significant reduction of the 24 hours pad test (mean 100.3 g; median 87.5, range 440 – 0; p < 0.01 Wilcoxon signed ranks test).

**Table 2:** Continence results in global and specific populations.

	Dry	Improved >50%	Improved <50%	Unchanged	Social continence	Substantial benefit (>50%)	
Global population (52)	16 (30.8%)	31 (59.6%)	4 (7.7%)	1 (1.9%)	**38 (73.1%)**	39 (75%)	(p < 0.05)
Mild incontinence (3)	3 (100%)	-	-	-	**3 (100%)**	3 (100%)	(p < 0.05)
Moderate incontinence (40)	12 (30%)	23 (57.5%)	4 (10%)	1 (2.5%)	**30 (75%)**	35 (87.5%)	(p < 0.05)
Severe incontinence (9)	1 (11.1%)	8 (88.9%)	-	-	**5 (55.5%)**	9 (100%)	(p < 0.05)
Radiotreated patients (6)	2 (33.3%)	3 (50%)	1 (16.7%)	-	**4 (66.7%)**	5 (83,3%)	(p < 0,05)
Previous surgery for SUI (30)	4 (13.3%)	23 (76.7%)	2 (6.7%)	1 (3.3%)	**19 (63.3%)**	27 (90%)	(p < 0.05)
Previous urethral surgery (15)	2 (13.3%)	12 (80%)	1 (6.7%)	-	**10 (66.7%)**	14 (93.3%)	(p < 0.05)

We had postoperative urodynamic data on 20 (38.5%) patients demonstrating in all of them a good bladder compliance (> 20 cmH_2_O / mL), absence of DO and significant bladder outlet obstruction (BOOI > 40). All patients evaluated had reached social continence and had no leakeage during urodynamic testing.

Taking into account the degree of preoperative incontinence, all patients (n = 3) with mild incontinence were dry. In patients with moderate incontinence (n = 40), 12 (30%) were dry, 23 (57.5%) improved ≥ 50%, 4 (10%) improved < 50% and only 1 (2.5%) unchanged; a total of 30 patients (75%) reached social continence. In patients with severe incontinence (n = 9), one (11.1%) was dry, 8 (88.9%) improved ≥ 50%; 5 patients (55.5%) achieved social continence (all p < 0.05 Fisher's exact test). Patients with mild incontinence had a 3.76 (95% CI 2.37 – 6) higher probability to be dry if compared to those with moderate and severe incontinence (p - value 0.03, Fisher's exact test).

In the subpopulation of patients with history of radiation treatment, 2 (33.3%) were dry, 3 (50%) were improved ≥ 50% and 1 (16.7%) had improved < 50%. A total of 4 patients (66.7%) reached social continence (all p < 0.05 Fisher's exact test). In the subpopulation of patients who have previously undergone surgical treatment for incontinence, 4 (13.3%) were dry, 23 (76.7%) improved ≥ 50%, 2 (6.7%) improved < 50%, and one (3.3%) was unchanged. In total, 19 patients (63.3%) reached social continence (all p < 0.05 Fisher's exact test). In the subpopulation of patients with previous urethral surgery, 2 (13.3%) were dry, 12 (80%) were improved ≥ 50% and 1 (6.7%) had improved < 50%. A total of 10 patients (66.7%) reached social continence (p < 0.01) (image 5) (all p < 0.05 Fisher's exact test).

Patients who underwent previous urethral surgery or previous incontinence surgery have a 65% lower probability to be dry if compared to patients without previous urethral or incontinence surgery (p - values < 0.05 and < 0.01, Fisher's exact test); they have a 2.23 higher probability to be > 50% improved without being dry (p - values < 0.05 and < 0.01, Fisher's exact test).

The average results of the quality of life questionnaire ICIQ - UI SF were 15.96 (median 16, range 12 – 20) before treatment and 7.7 (median 8, range 0 – 15) at the last follow-up, with a statistically significant variation (p < 0.01 Wilcoxon signed ranks test) (Table-3). The questionnaire on the subjective satisfaction of continence results (PGI - I) showed that 25 (49%) patients reported being very much improved, 17 (33.3%) much improved, 9 (17.6%) slightly improved; it is noteworthy that no patient expressed a negative subjective judgment towards treatment.

**Table 3:** Preoperative and postoperative ICIQ-SF questionnaire results.

Preoperative ICIQ-UI	Postoperative ICIQ-UI
17	6
17	13
16	7
17	7
14	9
18	13
16	11
20	9
15	9
15	10
16	11
17	7
15	8
17	12
15	8
19	8
16	12
15	8
14	7
16	7
16	0
14	9
19	15
19	9
19	0
15	6
16	9
15	0
15	9
15	0
15	0
17	10
14	6
15	7
17	9
17	6
16	7
12	9
18	9
15	11
15	5
15	5
13	10
17	7
15	8
16	11
18	8
15	1
15	11
15	8
16	6

## DISCUSSION

The results of our study, after a mean follow-up of 22.1 months, showed that 30.8% of patients were completely dry and 59.6% reduced losses > 50%: in total 73.1% reached social continence. These data are in line with published literature, although there are extremely variable results and definitions of continence; the results of dry rates vary from 92.3% of Gonzalez et al. on a small group of 13 patients (9), to 38.9% of Krause et al. (10). If we consider a substantial benefit (> 50%), we find values between 62.2% and 92.3%, confirming the validity of our results (10–14). Three studies have shown a significant reduction in ICIQ - SF score, as evidenced by our study (10, 14, 15).

The average operative time in our population was 51 minutes, in line with other literature studies (range 44 – 67.6) (9–15). The average number of device refillings was 1.55 with an average filling volume of 10.7 mL; other studies in literature show a greater number of refillings (range 1 – 4.5) and a higher average total cushion volume (17.5 – 18 mL) (11–14).

All studies in literature evidence a higher incidence of postoperative scrotal pain and paresthesiae (23.1 – 68.7%) (9, 11, 13, 15). Mühlstädt et al. showed a lower incidence of 5.6%, as they considered the persistence of scrotal pain 4 weeks after intervention (14). Using the same definition, in our series only 6 patients (11.5%) experienced scrotal pain and numbness. In our cohort we had a single case of superficial wound infection (2.3%), whereas in the literature the incidence of wound infection is variable between 4% and 19.4%, with rates of device explant for deep infection between 4% – 30.6%. This difference can be partially explained by the inclusion in previous studies of first - generation devices (9, 12–14). A careful preoperative perineal brushing with disinfectant solution, execution of hair removal before entering the operative room, adequate antibiotic prophylaxis with cephalosporins and a surgical technique that guarantees absolute sterility of procedures, have so far prevented from deep prosthesis infection requiring removal.

The most common complication encountered was port displacement (9.6%) resulting in the impossibility of modulating the filling of the cushion, requiring a surgical revision. In literature, Mühlstädt et al. indicate a rate of port erosion of 5.6%, but does not include cases of port displacement without surface erosion (14).

Results obtained by patients who underwent RT, urethral surgery or previous incontinence surgery, were slightly lower than the overall population. For the population of patients previously treated for incontinence, we have shown a significant reduction in the dry rate but not of the social continence. The reduced dry rate in patients who underwent previous urethral or incontinence surgery is balanced by a higher probability to have a > 50% improvement without being dry. These data suggest that ATOMS^®^ is not contraindicated in such complicated cases. Mühlstädt et al. also showed that RT patients achieved slightly worse continence results, but without any statistical significance (14). According to our results, we believe that the ATOMS^®^ system can be considered a first and second line surgical treatment for mild to moderate male SUI.

A study on the impact of ATOMS^®^ on the patient's sexual life showed a significant increase in the IEEF – 5 questionnaire values (16). In our study, the impact on the patient's sex life has not been directly analyzed, but we can hypothesize that overall improvement in the quality of life reported may also have a positive impact on the patient's sexual life.

One of the risks related to AUS is the development of urethral atrophy, that occurs on average in 7.9% of cases (1.9-28.6%) and appears to be linked to tissue hypoxia and previous radiotherapy. To reduce this possible complication, the ATOMS^®^ system is characterized by a non circumferential compression. Another possible complication, although rare, of the AUS is a mechanical failure in one component due to the complexity of the system, with rates varying from 2.0% to 13.8% (6). The lack of mechanical parts and the single - component built of the ATOMS^®^ system may thus represent an advantage, but we need a longer follow-up to confirm this hypothesis.

A comparative study of Chung et al. between adjustable slings (Argus) and non - adjustable slings (Advance) showed that social continence results are comparable (92% vs. 84%, p=0.45); however, when patients were left free to choose between the two devices, there was a tendency to choose the adjustable sling (57%) for the possibility of postoperative tensioning with a surgical procedure (17). A1ccording to international Guidelines, non - adjustable slings have consolidated indications, such as patients with moderate incontinence, non - irradiated, and with positive repositioning test. The advantage of ATOMS^®^ system is the office - based modulability, without surgical procedures, and the possibility to treat complex cases that are generally excluded by other slings.

We believe that this study has several strengths: the size of the cohort, an adequate follow-up, single surgeon interventions, the use of objective validated questionnaires. The main limitations of this study include the retrospective nature of the study, the lack of postoperative uorodynamic data for all patients, the lack of standardized follow-up visit schedule.

## CONCLUSIONS

In our experience the ATOMS^®^ system is an effective and safe surgical treatment of non - neurogenic male SUI. The results are good and are maintained in the follow-up. This system appears to be innovative for some unique characteristics: outpatient modulability and a single - incision transobturator technique. The implant is not contraindicated in patients who underwent urethral surgery and previous incontinence surgery.
